# Delivery of Theranostic Nanoparticles to Various Cancers by Means of Integrin-Binding Peptides

**DOI:** 10.3390/ijms232213735

**Published:** 2022-11-08

**Authors:** Elena A. Egorova, Maxim P. Nikitin

**Affiliations:** 1 Department of Nanobiomedicine, Sirius University of Science and Technology, 1 Olympic Ave., 354340 Sirius, Russia; 2 Institute of Experimental Oncology and Biomedical Technologies, Privolzhsky Research Medical University, 1 Meditsinskaya Str., 603081 Nizhny Novgorod, Russia; 3 Moscow Institute of Physics and Technology, 9 Institutskiy per., 141701 Dolgoprudny, Russia

**Keywords:** integrins, integrin-binding peptides, cyclic peptides, RGD peptide, gold nanoparticles, magnetic nanoparticles, quantum dots, liposomes, tumor targeting, photothermal therapy, hypothermia, cancer therapy

## Abstract

Active targeting of tumors is believed to be the key to efficient cancer therapy and accurate, early-stage diagnostics. Active targeting implies minimized off-targeting and associated cytotoxicity towards healthy tissue. One way to acquire active targeting is to employ conjugates of therapeutic agents with ligands known to bind receptors overexpressed onto cancer cells. The integrin receptor family has been studied as a target for cancer treatment for almost fifty years. However, systematic knowledge on their effects on cancer cells, is yet lacking, especially when utilized as an active targeting ligand for particulate formulations. Decoration with various integrin-targeting peptides has been reported to increase nanoparticle accumulation in tumors ≥ 3-fold when compared to passively targeted delivery. In recent years, many newly discovered or rationally designed integrin-binding peptides with excellent specificity towards a single integrin receptor have emerged. Here, we show a comprehensive analysis of previously unreviewed integrin-binding peptides, provide diverse modification routes for nanoparticle conjugation, and showcase the most notable examples of their use for tumor and metastases visualization and eradication to date, as well as possibilities for combined cancer therapies for a synergetic effect. This review aims to highlight the latest advancements in integrin-binding peptide development and is directed to aid transition to the development of novel nanoparticle-based theranostic agents for cancer therapy.

## 1. Introduction

Cancer therapy is typically perceived as a tremendous burden on a patient, not only affecting the tumor but all systems of the organism. Countless efforts were taken to develop a new approach to overcome this issue [1]. The most advanced approach recognized worldwide is the enhanced permeability and retention (EPR) [2,3,4]. The EPR effect or passive targeting to tumors is based on such physicochemical characteristics as size, shape, and charge of a therapeutic agent, whether it is a small molecule drug or a particulate formulation [5]. The EPR effect relies on pathophysiology of solid tumors, which involves defects of their vascular structure, impaired neoangiogenesis, and secretion of vascular mediators. For decades, this approach was a predominant topic of many publications devoted to new cancer therapies [3]. Thirty-five years after its discovery, the most tumor-selective and personalized method utilizing the EPR effect is the administration of oil-based formulations of nanodrugs via arterial infusion into tumor-supplying arteries [4]. EPR effect-based treatments also include various types of chemotherapy, photodynamic therapy, boron neutron capture therapy, etc. The main disadvantages of passive targeting of tumors are a high degree of off-targeting and its association with toxicity to the healthy tissue [1,6]. Passive targeting efficacy strongly depends on the tumor progression stage and requires a thorough tumor assessment. At the same time, the EPR effect has been widely studied in mice models, but the success of its translation into treatment of humans is still debated [7,8,9].

**Scheme 1:**
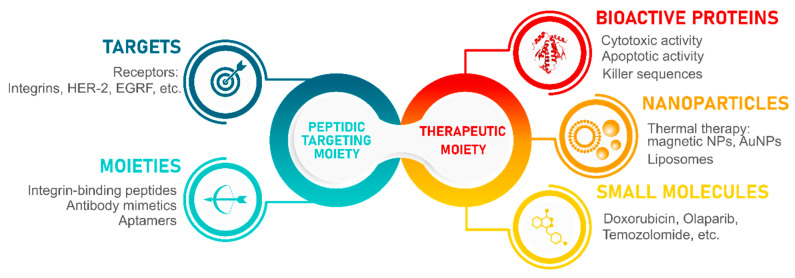
Cooperation of a targeting moiety known for a highly selective interaction with the target of interest and the anti-tumor effect of a therapeutic moiety is the basis of active targeting in cancer therapy. Thus, maximal efficiency and minimal off-targeting of such systems can be achieved. Examples of targets involved in the development of such anti-cancer formulations and their targeting ligands are partially described in this review and are given in the left part of the scheme. Examples of small-molecule cancer drugs, therapeutic nanoparticles, and bioactive proteins are given in the right part of the scheme. This review is mainly focused on integrin-binding peptides in combination with liposomes, gold and magnetic nanoparticles as the therapeutic agent of choice.

However, the targeting of tumors can be reinforced using active targeting [6]. In this way, the therapeutic agent is chosen from the passive targeting “toolbox”: a nanoparticle or a small drug molecule. The active targeting moiety is typically a ligand for a cellular receptor known to be present onto cancer cells with high abundance but not onto healthy cells (Scheme 1). Such moiety directs the conjugate to the tumor with a higher efficacy than passive targeting alone [10]. Integrin receptors are considered one of the key targets in cancer therapy [11,12]. Integrins are involved in many processes of the cell cycle: proliferation, migration, adhesion, and differentiation [13]. Integrins also play a pivotal role in tissue neovascularization [14]. Cancer cells show different patterns of integrin expression compared to healthy cells, which allows the tumor to progress [15,16]. More importantly, certain cancer types differ in this upregulation pattern. Integrin receptors are associated with multiple proteins of extracellular matrix (ECM), having given rise to many known peptide binders to integrins such as the RGD sequence (Arginine-Glycine-Aspartic acid) derived from fibronectin that mediates cell attachment. Other ECM proteins used to generate more integrin-binding peptides include: laminin, collagen, vitronectin, osteopontin, fibrinogen, and various cell adhesion molecules [17,18,19,20]. However, scrambled peptides or peptides derived from combinatorial libraries were also reported to bind integrins with high affinity [17,21,22,23].

Herein, most prominent examples of integrin-binding peptides as well as newly discovered and previously unreviewed sequences are described: a total of 22 peptide sequences specific for 14 different integrin receptors. Moreover, these peptidic targeting ligands are discussed as part of a therapeutic construct that consisting of targeting and therapeutic moieties. It is believed that highly selective interactions of these peptides with their targets are sufficient to precisely guide an intended therapeutic agent to tumors [24]. This way it could be possible to overcome undesired off-targeting and cytotoxicity to healthy cells though minimal non-specific interactions. To acquire a theranostic functionality, the following cargo could be used: small molecule drugs (Doxorubicin, Camptothecin, etc.) [25,26,27], radiolabels [28], as well as nanoparticles acting as drug carriers or capable of energy transformation into heat [29,30,31]. The main focus of this review is the use of soft nanoparticles, such as liposomes, and solid metallic nanoparticles (integrin-targeting quantum dots, gold nanoparticles, and magnetic nanoparticles). Such conjugates can be readily used in the visualization of tumors and metastases, as well as highly selective probes for photothermal therapy and hyperthermia, or as near-infrared (NIR) photosensitizers in radiotherapy.

## 2. Integrin Receptors

**Scheme 2:**
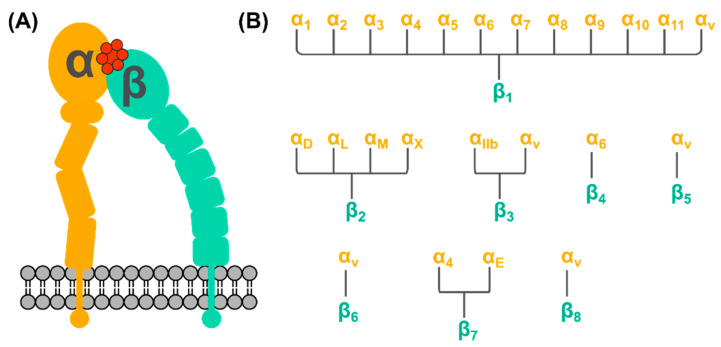
Generalized information on the structure of an integrin receptor (**A**) comprising two subunits α (in orange) and β (in cyan) bound to a peptide (in red). (**B**) There are 18 known α-subunits and eight β-subunits, that form 24 known integrin receptors. The β_1_ integrin receptor family is the largest and is targeted by most of the known integrin-binding peptides.

Integrins are transmembrane cellular receptors responsible for cell-cell adhesion [32]. When activated, they mediate processes of cell cycle regulation including cell proliferation, differentiation, migration, as well as the intracellular cytoskeleton reorganization, and the movement of newly recruited receptors to the cell membrane [13]. Integrin receptors comprise two subunits, a combination of one out of 18 possible α-subunits and one out of eight possible β-subunits (Scheme 2). There are 24 known integrin receptors and half of them belongs to the β_1_ superfamily. One cell can express multiple integrin receptors to maintain its normal function [32]. At the same time, one integrin receptor can bind multiple ligands, and one integrin-binding ligand can bind multiple integrin receptors.

Integrins mediate many important processes in the cell cycle and their malfunction is usually associated with cancer. Deregulated integrin signaling permits unrestrained cancer cell proliferation and invasion in healthy tissues leading to tumor formation and further development [12]. Possibly, this also allows for the survival of cancer cells in the microenvironments upon their migration and tumor neovascularization [33]. This deregulation involves both upregulation and downregulation of integrins expression. For example, upregulation occurs when adhesion of cancer cells takes place to facilitate the tumor growth, neovascularization, and metastasis; and downregulation occurs when cancer cells detach to migrate. The growing evidence suggests that certain cancers exhibit selective integrins expression or their overexpression [11,15,34]. For example, tumor vascular endothelial cells (tVECs) highly express α_v_β_3_ and α_v_β_5_ integrin receptors [35]. A comprehensive study by Arun et al. screened 17 different solid tumor types for integrin expression profiles and identified suitable cancer-type specific targets [36]. In some cancers, such as glioblastoma multiforme, the overexpression of some integrin subunits was estimated to reach a ~10-fold difference in comparison to normal tissue. No potential integrin targets were found for the following five tumors: urothelial bladder carcinoma, breast invasive carcinoma, paraganglioma and pheochromocytoma, prostate adenocarcinoma, and rectum adenocarcinoma. Additionally, it was possible to identify a single integrin overexpressed in a specific cancer: α_E_β_7_ in the three studied varieties of kidney cancer. At the same time, the following ten integrins were not overexpressed in the studied 17 solid tumors: α_D_β_2_, α_L_β_2_, α_M_β_2_, α_X_β_2_, α_4_β_7_, α_1_β_1_, α_10_β_1_, α_11_β_1_, α_9_β_1_, and α_8_β_1_. However, these findings do not indicate that these integrins can be considered absolutely cancer-unrelated, as more analysis is needed in order to claim so.

Due to multiple lines of evidence showing that integrins are cell-type specific and due to a high extent of their involvement in cancer, they became a highly alluring target for cancer therapy.

## 3. Integrin-Binding Peptides

### 3.1. General Overview of Integrin-Binding Peptides

**Scheme 3:**
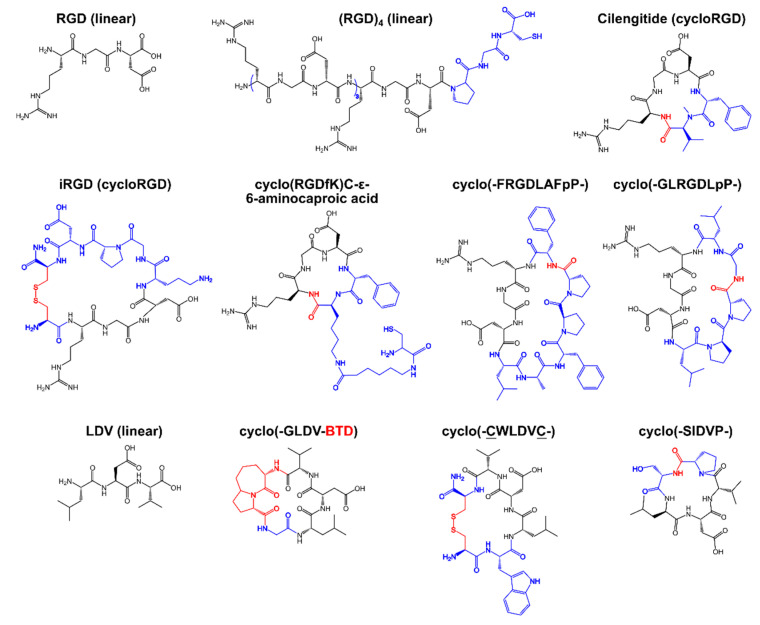
Chemical structures of integrin-binding peptides based on the sequences of the RGD and LDV peptides. Color code: the original peptide sequence of the predecessor peptide is indicated in black; residues not present in the predecessor sequence are indicated in blue; and conjugation groups for cyclic peptides are indicated in red. BTD—β-turn dipeptide.

**Scheme 4:**
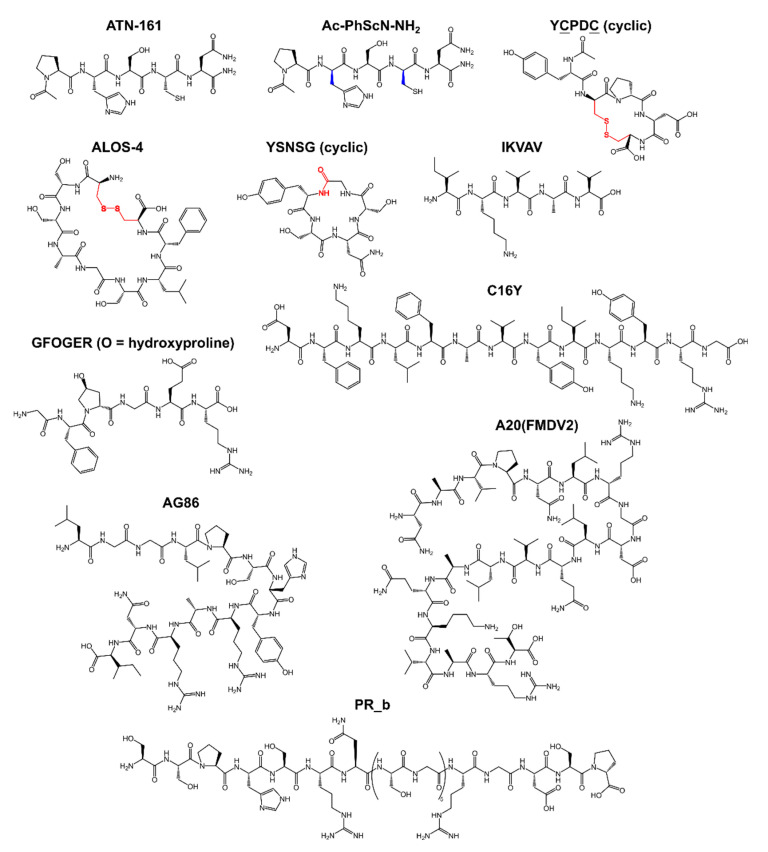
Chemical structures of non-RGD based integrin-binding peptides. Color code: the original peptide sequence of the predecessor peptide is indicated in black; D-residues are indicated in blue; and conjugation groups for cyclic peptides are indicated in red. O—hydroxyproline.

Integrins bind endogenous ligands such as vitronectin, fibronectin, fibrinogen, osteopontin, collagen, laminin, and different adhesion molecules expressed onto vascular, mucosal and any other cells known to interact through integrin binding [37]. Binding sequences of these proteins gave rise to so-called integrin-binding, or integrin-targeting, peptides, the most famous example of being the RGD peptide. The RGD peptide derives from fibronectin and is a broad-spectrum antagonist of multiple integrin receptors from the β_1_ superfamily (Table 1, Scheme 3 and Scheme 4).

### 3.2. Development of Integrin-Binding Peptides

The RGD peptide and its derivatives have been under development since the 1980s and were used in the treatment of advanced solid tumors, breast cancer, glioblastoma, pancreatic cancer, colon cancer, glioma, melanoma, and renal cell carcinoma. However, the linear peptide sequence has exhibited a clinically poor performance (IC_50_ = 87 nM), low stability, and as a consequence short half-life, poor selectivity towards specific integrin receptors, and low tissue penetration [73,74]. Cyclization of the RGD peptide resulted in Cilengitide (the proprietary name by Merck) and other cyclic peptides based on it. These ligands were reported to exhibit improved stability in a physiological environment, a more stable peptide-integrin interaction, as well as marginally increased specificity towards α_v_β_1_, α_v_β_3_, and α_v_β_5_ integrins [75]. This selectivity can also be expressed in terms of binding inhibition for known protein integrin binders. For example, Cilengitide showed a 1500-fold higher activity for inhibition of vitronectin binding to α_v_β_3_ than fibrinogen binding to α_IIb_β_3_ [39]. This difference in binding affinity between different integrins is key to selective targeting by means of integrin-binding peptides.

LDV, another minimalistic peptide sequence deriving from fibronectin, is known to target β_1_, in particular α_4_β_1_, and α_4_β_7_ (Table 1) [49]. Its linear form and the parent EILDV sequence were used in several cell adhesion studies, including human melanoma [49,50]. It was found that inclusion of D-amino acids in the parent sequence improved anti-metastatic properties in vivo [49]. Concurrently, this short motif was reported as part of cyclo(-CWLDVC-), a cyclic hexapeptide inhibiting α_4_β_1_, vascular cell adhesion molecule-1, and mucosal adhesion cell adhesion molecule-1 [51]. This discovery was the first evidence of the three receptors having a shared binding motif. Several cyclic variants of LDV were reported: cyclo(-SlDVP-) [52] and cyclo(-GLDV-BTD), where BTD stands for β-turn dipeptide [53]. Although several cyclic and D-amino acid derivatives of LDV showed at least 100-fold higher potency over the linear peptide [51,76], they are not as widely used in anti-cancer formulation development as derivatives of RGD [77]. One possible reason may be that α_4_β_1_ (or Very Late Antigen-4, VLA4) is abundantly present in immune competent cells. The role of VLA4 in cancer progression has been strongly associated with the activation of fibronectin isoforms. A recent report showed that in a mouse model of colon adenocarcinoma depleted of α_4_β_1_ integrin, an accelerated tumor growth was observed [78]. Another recent finding suggests that the blockade of α_4_β_1_ sensitizes leukemic and myeloma tumor cells to immune therapy in vitro [79]. Thus, LDV-derived integrin-binding peptides are attractive agent for cancer research, especially in hematological malignancies.

When finished, the clinical trials of Cilengitide^®^ revealed that its efficacy largely depended on the cancer type, and its commercial development was discontinued [38]. This outcome aspires a logical question: what is more beneficial for cancer therapy, a multivalent integrin binder, or a binder showing predominant binding to only one integrin? Consequently, new integrin-binding peptides and new integrin targets were proposed.

One group of new peptides that only bind one preferred integrin receptor includes Cilengitide modifications with incorporated substitutions for unnatural lipophilic amino acids. These antagonists show higher selectivity for α_v_β_3_, which is involved in angiogenesis, over other integrins [54]. The RGD motif is present in other proteins apart from fibronectin, for example, in the envelope protein of foot-and-mouth disease virus. Through rational design, two cyclic peptides were identified to predominantly bind either α_v_β_6_ or α_v_β_8_: cyclo(FRGDLAFpP) and cyclo(GLRGDLpP), respectively [47,48]. α_v_β_6_ integrin is known to be overexpressed in certain cancers: colon, lung, cervix, ovaries/fallopian tube, pancreas, head and neck cancer (human oral squamous cell carcinoma in particular), triple-negative breast cancer [80,81,82]. To this end, very little is known about the pathophysiological role of α_v_β_8_ apart from its involvement in the cellular entry of viruses, development of cancer and autoimmunity, but with the help of this newly developed peptide, it can be unraveled. Based on the data obtained in the human melanoma model, it was suggested that α_v_β_8_ exhibits very low expression on the surface of healthy cells and tissues, while in cancer cells it is elevated [48]. It is known that α_v_β_8_-positive cancer stem cells are present in glioblastomas and ovarian carcinomas [83,84,85]. Moreover, β_8_ integrin regulates angiogenesis and tumor cell invasiveness to a great extent. The discrimination between different integrin receptors is of great importance for the development of anti-cancer therapeutics as it can assist in development of new and/or personalized cancer therapy.

More RGD-bearing peptides were reported for their excellent performance: multimeric (RGD)_4_ and iRGD. iRGD is cyclized via the side chains of two terminal cysteines in the sequence of cyclo(-CRGDKGPDC-) [40,41]. Compared to both linear and cyclic RGD peptides, iRGD possesses higher affinity for α_v_ integrins: the mid- to low-nanomolar range [86]. The CRGDK fragment binds to α_v_ integrins and neuropilin-1, which allows for deeper penetration into the tumor parenchyma. Neuropilin-1 is the coreceptor to vascular endothelial growth factor, the key mediator of angiogenesis in cancer [87,88]. Thus, iRGD is considered the most powerful RGD-based integrin binder in cancer therapy of multiple cancers to date [89]. Another cyclic RGD variant, cyclo[RGDfK(C-ε-6-aminocaproic acid)] was especially designed for coupling onto nanocarriers such as micelles of micellular polyplexes [42]. Interestingly, the reported cycloRGD-decorated micelles were shown to accumulate in the perinuclear region of HeLa cells rich in α_v_ integrins, which was an important finding for development of these targeted gene delivery systems [44,45,46].

A lesser reviewed sequence, the PR_b peptide is a conjugate of two distinct motifs found in fibronectin, PHSRN and RGDPS, coupled via a (SG)_5_ spacer [19,70]. Due to its synergistic design, PR_b exhibits high affinity to α_5_β_1_ integrin [90]. Interestingly, this integrin-binding peptide was originally proposed as an amphiphilic molecule capable of incorporation into lipid bilayers. Since then, it was only used as a targeting component in liposomes for cancer cell-selective gene delivery [91,92].

Another fragment from the foot-and-mouth disease virus envelope protein was identified as a selective integrin binder [71]. A 20-residue long fragment, A20FMDV2, showed K_d_ = 0.22 nM for α_v_β_6_ (for the sequence, see Table 1) [72,93,94]. This longer peptide suffers from short plasma half-life. This obstacle was overcome by either PEGylation (PEG—poly(ethylene glycol)) [95], via cyclization [96], or by substitution of certain residues in the parent sequence [96,97,98]. A20FMDV2 is extensively used in imaging studies with positron emission tomography (PET) in vivo models of pancreatic [99,100] and lung cancer [93]. Additionally, the safety of radiolabeled A20FMDV2 was shown in humans [28,101].

Recently, non-RGD integrin-targeting peptides have started to attract more and more attention. For example, ATN-161 peptide (sequence Ac-PHSCN-NH_2_, or just PHSCN, Table 1) binging α_v_β_3_ and α_5_β_1_ was mapped in the sequence of fibronectin [102]. This peptide is now proposed to be used in Sars-CoV-2 therapy [103,104]. Apart from viral infection therapy, ATN-161 was shown to inhibit angiogenesis [55], reduce tumor growth and tumor recurrence both in prostate and breast cancer models [56,57], as well blocking cancer invasion, lung colonization and bone colony progression [57]. In Phase I clinical trials, this peptide was used in systemic monotherapy in patients with solid tumors and prevented disease progression for up to 14 months [59]. As other integrin-binging peptides, PHSCN was subjected to modification in order to increase its binding efficacy. It was performed through substitution of L-histidine and L-cysteine for their D-counterparts. The resulting Ac-PhScN-NH_2_ peptide showed a superior therapeutic effect in a breast cancer model—IC_50_ in the pg/mL (sub μM) regimen [57]. Unlike other integrin-binding peptides, PHSCN utilizes the side chain of its Cysteine residue to maintain the integrin binding [58], while other peptides use hydrogen bonding, π-π stacking, hydrophobic and potentially electrostatic interactions [48]. However, this unique feature can be a disadvantage: depending on the setup, reducing agents in the system can affect the peptide binding. Moreover, one must be cautious of the thiol chemistry when devising a coupling strategy for this targeting peptide to a carrier moiety or a small-molecule drug. Although it has to be noted that PHSCN was not found to be prone to dimerization through the cysteine side chain [58].

The ALOS-4 peptide (sequence cyclo(-CSSAGSLFC-)) was selected through phage display and does not bear any resemble with natural integrin-binding peptides or proteins [23]. Its known target is α_v_β_3_ integrin, which is overexpressed in melanoma, glioma, prostate, and breast cancer cells [11]. Although ALOS-4 binding to α_v_β_3_ was found to be somewhat weaker than desired for clinical use—K_d_ = 2.55 ± 1.22 μM, the effect of ALOS-4 was comparable to that of a known antibody inhibitor of α_v_β_3_. In the mouse melanoma model at the dose of 0.3 mg/kg, the following effects were observed: (i) daily ALOS-4 administration doubled the time needed to establish a tumor in mice subcutaneously inoculated with B16F10 cells compared to untreated mice; (ii) the tumor size was 3–5-fold smaller for treated mice; (iii) and the number of metastases in lungs was reduced by 87%. Next, ALOS-4 was tested in a subcutaneous xenograft model of A375 human melanoma as a single administration one-day post inoculation [60]. The obtained results supported the previous findings: ALOS-4 modulates α_v_β_3_ signaling leading to metastatic arrest, is well-tolerated, and exhibits a prolonged plasma half-life time. However, by itself, ALOS-4 did not express any cytotoxicity towards cancer cells as it had no effect on cancer cell proliferation nor viability. The conjugate of ALOS-4 and an anti-cancer drug Camptothecin did induce Caspase 3 activation, thus causing apoptosis of human metastatic melanoma WM-266-4 cells [26]. Since the ALOS-4 peptide was first reported in 2016, available data on this peptide is limited. However, there are all prerequisites for it to become an extremely potent integrin binder if sequence modification and optimization would be conducted.

Another yet not fully assessed integrin-binding peptide is C16Y (sequence DFKLFAVYIKYR) [17,21]. It is a scrambled peptide based on a fragment of laminin and it binds α_v_β_3_ and α_5_β_1_ integrins. The C16Y peptide blocks angiogenesis and tumor growth in vitro [21]. Similar to ALOS-4, C16Y was used in combination with a small-molecule anti-cancer drug cisplatin increasing its efficacy up to 5-fold in inducing apoptosis of ovarian cancer cells [105]. Conjugation of C16Y with a lipophilic construct yielded spherical nanostructures self-assembled at physiologic conditions, but dissociated into individual peptides when in weakly acidic tumor microenvironment [106]. Similarly designed C16Y amphiphiles were used as a targeting moiety in conjugation with liposomes [107]. These lipid-based C16Y formulations were capable of effective targeting and delivery of a therapeutic component to tumors in vivo.

YSNSG cyclopeptide derived from tumstatin also binds α_v_β_3_. It was shown to inhibit melanoma progression in vivo [61] and tumor neovascularization in vivo [62]. It was determined that this peptide penetrated solid tumors [63]. Furthermore, in an in vitro model it was shown that YSNSG did not affect endothelial cell proliferation but inhibited cell migration by 83% [62]. This evidence indicates that the YSNSG cyclopeptide is a good candidate for further development of targeted delivery platforms.

Cyclic YCPDC peptide demonstrates IC_50_ of 0.1 nM and >1000-fold higher specificity towards α_4_β_1_ over other β_1_ integrins [64]. This peptide was obtained through rational design based on the RCDPC motif. YCPDC is known to specifically bind to various multiple myeloma cells [108], and has been used as a targeting moiety in lipid-based treatment for this disease [109]. The potential of this peptide is yet to be fully investigated.

Another promising peptide, AG86 (sequence of LGGLPSHYRARNI) was found in the laminin sequence in 2010 and identified as a high-affinity binder for α_6_β_4_ integrin [69]. Preliminary affinity of this interaction was found to be 2.5 ± 0.9 μM [92]. However, according to the same method, the PR-b peptide affinity to α_5_β_1_ was found to be f 5.0 ± 3.2 μM, drastically different to the value of 76.3 ± 6.3 nM obtained with a standard ELISA assay [110]. Nonetheless, AG86 was successfully used as a targeting moiety in liposomal formulations to HVP-18 positive cervical cancer cells [91,92].

Additionally, there is a group of integrin-binding peptides worth mentioning. GFOGER peptide is capable of binding α_11_β_1_ integrin, whereas IKVAV binds to α_3_β_1_ and α_6_β_1_ (Table 1). GFOGER has been used for modulation of transplanted human bone marrow-derived mesenchymal stem cell survival, engraftment, and reparative activities [18,20]. The laminin-derived IKVAV is known to be important for cell attachment and migration, as well as for neurite outgrowth [67]. These peptides are essential for regenerative medicine, tissue repair, applications aiming to enhance cell integration, differentiation, or maturation, such as in spheroid culturing. In the latter case, through chemical coupling these peptides are incorporated in a biocompatible hydrogel matrix that the cells of interest are exposed to [20,37,111,112,113]. Apart from regenerative medicine, GFOGER and IKVAV are typically used for studying migration and other process associated with cancer [114] or for creating in vitro models to resemble the tumor microenvironment [66,68,115]. It was shown that glioma stem-like cells exposed to supramolecular fibers with IKVAV incorporated, undergo apoptosis via inhibition of focal adhesion kinase combined with inhibition of epidermal growth factor receptor (EGFR) [116]. These fibers act as a surrogate extracellular matrix, block the trafficking of activated β_1_-integrins, impair the signaling, and thus induce cancer cell apoptosis. However, if administrated alone, the IKVAV peptide and another laminin-derived integrin-binding peptide C16 (peptide sequence KAFDITYVRLKF) cause tumor progression [17]. These peptides provide good targeting, but possible and undesirable effects have to be balanced out through the use of an additional moiety. This moiety has to provide a therapeutic effect: cytotoxicity, switching to apoptosis or necrosis, etc. These include radiotherapy agents, small molecule anti-cancer drugs, bioactive proteins, or nanoparticles, which this review is focused on. Even the above-mentioned IKVAV supramolecular fibers could be considered as such moiety [116].

To conclude, there are known and thoroughly investigated peptides binding multiple integrins and newly discovered peptides predominantly binding one integrin receptor. In many cases, to improve affinity or achieve the selectivity of integrin binding, peptide sequence optimization is required. It can be cyclization via the peptide backbone or via the side chain of additional residues (for example, terminal cysteines or a β-turn forming domain), substitution for D-isomeric amino acids, or substitution for peptidomimetic residues. These manipulations to the chemical structure alter a molecule’s geometry and physicochemical properties allowing for a more stable fitting in the integrin binding pocket, as well as an increased plasma half-life (Table 1). Additionally, some of the integrin-binding peptides exhibit anti-cancer activity by themselves (for example, ATN-161), whereas others either do not have an effect or even induce faster tumor progression if administered alone (for example, IKVAV). Moreover, a thorough assessment in in vivo cancer models is needed for the majority of the newly-developed integrin-binding peptides. However, there are many promising peptide sequences that can be employed as a targeting moiety for new biomaterials in cancer research.

## 4. Conjugation Strategies for Integrin-Binding Peptides

Nanoparticles are very diverse in their nature; lipid-based or metallic, soft or solid, they provide a great choice of features adaptable to an intended application. Liposomes are highly biocompatible and well-tolerated, capable of carrying significant payloads of both hydrophilic and hydrophobic compounds [117]. However, the derivatization of liposomes with a targeting peptide can be problematic. Often, the surface availability of the targeting or biologically active peptide necessary for its proper functioning is compromised by interactions with the lipids, resulting in the peptide engulfment into the bilayer [118]. For display purposes, it is more common to use solid metallic or metal oxide nanoparticles, such as magnetic nanoparticles (MNPs) or gold (AuNPs) ones.

**Scheme 5:**
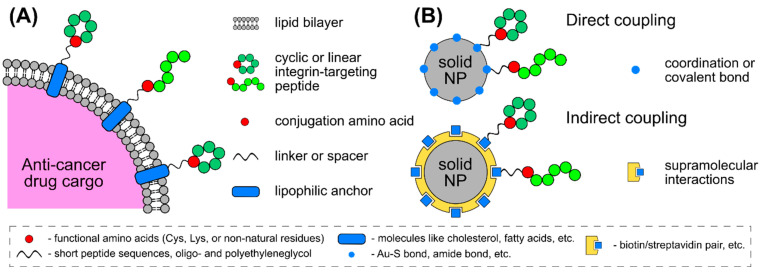
Integrin-targeting peptide coupling strategies for (**A**) soft nanoparticles such as liposomes and (**B**) solid polymer- or metal-based nanoparticles. The peptides are equipped with a conjugation residue that is optionally attached to a surface via a flexible linker or spacer. Anchoring to a surface is mediated by conjugated lipophilic moieties in the case of liposomal delivery system or by direct or indirect coupling in the case of solid nanoparticles. The box contains examples of chemical blocks used for this purpose.

Before going further, we would like to briefly discuss coupling strategies frequently used to conjugate an integrin-binding peptide to a nanoparticle surface. Peptides are easily subjected to incorporation of functional groups suitable for chemical ligation (Scheme 5). Ligation can affect the peptide’s activity, thus the use of linkers such as oligo-glycine (G_n_; typical *n* ≥ 2) [107,119], serine-glycine repeats ([SG]_n_, typical *n* ≥ 2), or oligo-ethyleneglycols ([-CH_2_CH_2_O]_n_, typical *n* ≥ 4) [118,120,121,122,123,124,125], has to be considered. However, in some cases, a peptide is coupled to the carrier nanoparticle via its N-terminus (or any other primary amine in the sequence) [119], or it can be simply integrated into the existing peptide sequence of a protein/peptide carrier [112,116]. The most popular way is to incorporate a unique lysine or cysteine residue into the peptide sequence to perform the ligation on their side chains. Thus, the lysine can be coupled to a carboxylic acid displayed onto the nanoparticle surface [47,48,91,92,121]. A similar method is used to couple peptides to anionic polymers or nanoparticles coated with these polymers [126,127]. Likewise, the cysteine can be coupled to a displayed maleimide [107,120,122,128] or to a free amine group via sulfo-SMCC coupling [129]. The addition of cysteine or any other thiols is typical for peptidic modification of AuNPs, as it allows for the formation of the strong Au-S bond [130,131,132]. As for lipid-based formulations, lipidation of peptides allows for their incorporation into lipid bilayers or micelles. Typical lipids used for this purpose are cholesteryl hemisuccinate [118,123], charged lipids with an available primary amine or carboxylic group [120,133] or simple coupling of an acyl fatty chain (C16, C18, etc.) [91,92,134,135]. “Click” chemistry is also utilized for peptide coupling to nanoparticles [136,137,138]. The incorporation of peptides via non-covalent forces is also possible. Supramolecular interactions are directional, whereas chemi-/physisorption is more chaotic and may affect the peptide conformation and binding ability, however the latter is suitable for protein immobilization [139]. The examples include the use of the biotin/streptavidin pair and the coiled-coil mediated display [140,141]. Multimerization of an integrin-binding peptide can also be achieved through its conjugation to serum albumins and subsequent chemisorption of this conjugate onto a nanoparticle surface [142,143,144].

The work by Stefanick et al. is an example of a thorough work conducted to optimize the composition of targeting nanoparticles [109]. In this study, VLA4pep targeting the α_4_β_1_ integrin (YCPDC) was tethered to a dipalmitoyl chain via a composite spacer. This spacer was conjugated to the peptide N-terminus and comprised a short diethylene glycol unit (EG_2_) coupled to an oligo-lysine domain, which was followed by another oligo-EG spacer of variable length (EG_N_). This amphiphile was incorporated into PEGylated liposomes. The presence of oligo-lysine allowed to modulate hydrophilicity of the resulting liposomes. At the same time, the effect of the oligo-EG-spacer length was anticipated to determine the targeting peptide presentation and availability. The linker length, the number of extra lysines, the peptide display density, and the liposome size were varied in order to determine their effects on in vitro and in vivo uptake by cancer cells (NCI-H929 and MM.1S myeloma cells).

In that study, it was found that the extra lysines presence was essential for the targeted liposome cellular uptake. When the other variables were fixed to certain values, only the liposomes with three or four extra lysines showed elevated cellular uptake, while no lysine, one, or two—did not show any significant impact. Concurrently, the cellular uptake did not differ between the three and four lysines cases. This indicated that the extra three lysines were required for VLA4pep to be properly displayed and not to be compromised by interactions with the carrier itself. PEG with MW 2000 Da is a typical component of PEGylated liposomes, but using the same PEG (EG_45_) as the spacer resulted in approximately seven-times less efficient cellular uptake compared to a PEG with MW 264 Da (EG_6_) spacer. Excessive flexibility can be enemy to precision of peptide presentation. The targeted PEGylated liposomes were also prepared in three sizes: 30, 50, and 100 nm in diameter. The 30 nm liposomes were the least effective regardless of the oligo-EG spacer length. However, the 50 nm and 100 nm liposomes were taken up more efficiently, but the optimal spacer length differed between the two cases. The best uptake for the 100 nm liposomes was obtained with the EG_6_ spacer, while for the 50 nm liposomes it was the EG_12_ spacer. This indicated that the optimal spacer length depended on the surface curvature as it affects the peptide presentation. The peptide display density topped off at 2% (mol.) with almost doubled cellular uptake efficiency compared to the 1% (mol.) density. However, the in vivo tumor accumulation of these liposomes did not follow the conclusions drawn from the in vitro uptake study. According to the in vivo evaluation, the best performance was exhibited by the 50 nm liposomes with the EG_6_ spacer and 0.5% peptide display density, however the overall level was decreased in comparison to the in vitro results.

In another study, the display density of cyclo[RGDfK(C-ε-6-aminocaproic acid)] was found to be crucial for the cellular uptake and cytotoxicity of oxaliplatin-loaded micelles (~30 nm in diameter) in U87MG human glioblastoma cells [43]. Significant oxaliplatin doses were delivered in vitro for the 20% peptide density, while there was significant improvement for the 40% peptide density.

Combined, these findings emphasize that the rational design and thorough optimization are necessary for efficient anti-cancer peptide-targeted nanoparticle formulations.

## 5. Integrin-Binding Peptides as Targeting Moieties in Nanoparticle Formulations

**Scheme 6:**
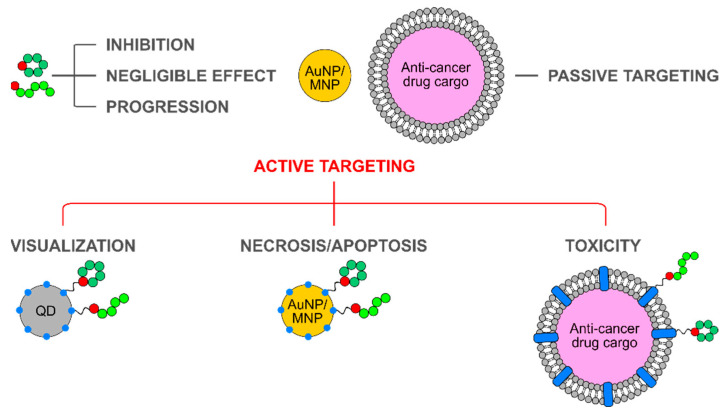
Active targeting to tumors by integrin-binding peptides combines the effects deriving from the peptides and a carrier nanoparticle. Integrin-binding peptides show various effects on cancer cells and tumors: from inhibition of tumor progression and metastasis to the opposite effect, and in some cases no effect on cancer cell proliferation or cytotoxicity are observed. If administered in a non-targeted way, nanoparticles retain in tumors due to the EPR effect, or passive targeting. Combined together, these active targeted nanoparticles retain in tumors due to both active and passive targeting, and are proposed for tumor and metastases visualization and eradication of thereof due to cytotoxicity, necrosis, or apoptosis.

Active targeting of tumors by means of nanoparticles allows for higher levels of accumulation of therapeutic agents, minimized toxicity caused by off-targeting and non-specific interactions. Apart from the targeting ability, integrin-binding peptides show varying effects: from inhibition of tumor progression and metastasis to the opposite, and in some cases no effect on cancer cell proliferation or cytotoxicity is observed (Scheme 6). For the purpose of active targeting, the integrin-binding peptide of choice must show good tumor-homing properties, however its therapeutic effect or the lack thereof can be considered secondary. Nonetheless, a thorough assessment of integrin-targeting nanoparticles is needed to prove this point. With the large number of newly discovered integrin-binding peptides, this task is still of great interest.

The use of carrier nanoparticles pursues several goals. As free peptides are cleared quickly, protection from degradation and prolongation of plasma half-live are desired and easily achieved through coupling to a nanoparticle surface. Conjugation provides peptide multimerization and increases avidity to a corresponding integrin by at least two orders of magnitude [125,129]. It was also reported that this effect is strongly particle size-dependent, which can be due to the EPR effect, or passive targeting. Nanoparticles can be designed in a way to perform a therapeutic effect due to the presence of a cytotoxic cargo, for example, in the case of liposomes or polymersomes. Or it can be acquired through photothermal therapy (PTT) or hyperthermia, when AuNPs and MNPs are involved. Such active-targeted formulations retain in tumors due to both active and passive targeting and are proposed for tumor and metastases visualization and eradication thereof due to cytotoxicity and induced necrosis/apoptosis. On the other hand, such targeted systems can aid the investigation of integrin receptor roles in various infectious, autoimmune, and inflammatory diseases.

### 5.1. Lipid-Based Particles: Liposomes and Micelles

Liposomes comprise a lipid bilayer confined to a spherical or near-spherical shape. Lipids are usually composed of acyl chains attached to a headgroup. The bilayer is organized in such a way that the hydrophobic alkyl chains are sequestered from the aqueous environment and are located between the hydrophilic headgroups that point outwards and are exposed to the environment. Liposomes are highly biocompatible, exhibit low cytotoxicity and are suitable for encapsulation of inorganic, hydrophobic, and hydrophilic therapeutic agents [117,145].

Successful decoration of liposomes with integrin-binding peptides was reported: the target size of liposomes is typically 100 nm, although choices of smaller or larger sizes were also described [107,109,146]. Displayed peptides preserved their targeting properties both in vitro [120,121] and in vivo [122], concurrently allowing for better tumor accumulation and lesser off-targeting [108,122,147]. Herein, we focus on decoration of liposomes with non-RGD peptides. For more information on RGD and cyclic RGD peptide-modified liposomes, the reader is kindly referred to Cheng et al. [146].

Loading liposomes with anti-cancer drugs such as Doxorubicin (DOX) provides the peptide-decorated liposomes with a cytotoxic effect that is only performed when the liposomes are taken up by cancer cells overexpressing the target integrin in vitro [121] and in vivo [108]. Furthermore, loading liposomes with DOX increased its bioavailability, while decoration with ATN-161 peptide decreased the IC_50_ value by 56%. However, liposomal formulation has to be chosen wisely: liposomes can leak and thus the cytotoxic payload is partially lost causing off-target induced cytotoxicity. The same peptide was displayed on the surface of reduction-sensitive polymersomes loaded with DOX [147]. The study in the B16F10 melanoma model showed that DOX accumulation in the tumor was 2.5 times higher for the ATN-161 group compared to polymersomes that only contained DOX. Similarly, more efficient tumor inhibition post inoculation (the tumor volume was three times smaller) and an increase in the survival rate (the median survival time of mice increased by 69%) were observed for the targeted group.

Another promising peptide C16Y, known to block angiogenesis and progression in vitro [21], has been modified with lipophilic moieties in order to create lipid-based targeted systems. It was shown that its conjugation to DSPE-PEG2000 did not affect its activity and allowed for active α_v_β_3_ targeting of the C16Y-bearing liposomes [107]. Conjugation with four molecules of l,3-diethylaminopropyl isothiocyanate (DEAP) yielded spherical nanostructures self-assembled at physiologic conditions, but dissociated into individual molecules when in the weakly acidic tumor microenvironment [106]. These nanoprobes were loaded with a hydrophobic dye allowing for MDA-MB-231 tumor imaging in vivo, as well as with DOX for combination chemotherapy. In 4T1, a highly metastatic murine breast cancer model, the DEAP-C16Y conjugate showed almost a halved tumor weight, mean tumor vessel number, and number of metastases in the lungs relative to the C16Y peptide alone. Furthermore, the DEAP-C16Y conjugate loaded with DOX was more effective than free DOX, and further improved tumor and metastasis inhibition compared to DEAP-C16Y by ~50%.

An interesting approach was proposed by Wei et al.: DOX-loaded liposomes were decorated with a cleavable peptide, that would only release Cilengitide in the tumor microenvironment, thus modulating the tumor angiogenesis to improve blood perfusion and, consequently, drug delivery [135]. This cleavable peptide is cleaved by membrane type 1 matrix metalloproteinase, highly expressed in tumor endothelial cells. This approach was tested in a pancreatic cancer model in BxPC-3-bearing mice. Significant tumor growth inhibition and increased tumor cell apoptosis were detected: more than a doubled effect in contrast to non-targeted DOX-liposomes. Furthermore, non-cleavable Cilengitide liposomes did not show any significant difference from a negative control. Similarly, VLA4pep (or YCPDC) was conjugated to lipid-based micelles containing DOX via an acid-labile bond [108]. This approach was chosen to prevent the premature DOX release and thus nonspecific toxicity. Studies in NCI-H929 multiple myeloma tumor model showed that targeted delivery of DOX inhibited the tumor progression more effectively than non-targeted delivery. The tumor volume in the non-targeted group was twice of that in the targeted group. Additionally, treatment with the same equivalent of free DOX led to a great level of systemic toxicity causing moribundity only after 7 days of treatment. Compared with free DOX and non-targeted DOX-micelles, DOX accumulation in the tumor for the targeted group reached up to ~10- and ~5-fold higher levels, respectively. Another important aspect of integrin-targeting micelles is that they not only target cancerous cells but also tumor neovasculature abundantly expressing integrin receptors. The Kataoka group devised several highly efficient platforms for anti-angiogenic gene therapy through tumor vascular targeting using cycloRGD-appended polyplex micelles loaded with plasmid (p)DNA encoding a soluble form of VEGF receptor-1 [45,46].

The AG86 peptide is a laminin-derived peptide discovered in 2010 known for binding α_6_β_4_ integrin [69]. The information on this peptide is very limited; however, this peptide was already used for targeted delivery by means of liposomes [91,92]. It was shown in vitro that these liposomes were only targeting HPV-18 positive HeLa cells and not the control “healthy” C33A cells [91]. Moreover, the targeting efficacy of one integrin-binding peptide towards a specific cell line was modulated by introducing a second integrin-targeting peptide. A combination of AG86 and the PR_b peptide allowed such targeted liposomes to discriminate between the cells expressing both targets (α_6_β_4_ and α_5_β_1_, respectively) from the cells expressing high levels of only one of said integrins [92]. The dual targeting can also be performed though using two targeting moieties of different nature. For example, the targeting capacity of RGD-decorated liposomes in a triple-negative breast cancer model was enhanced through simultaneous display of fructose both in vitro and in vivo [123]. This heteromultivalency may allow for minimal off-targeting and a more precise and personalized approach to cancer therapy.

### 5.2. Metallic Nanoparticles

#### 5.2.1. Quantum Dots

Quantum dots (QDs) are semiconductor nanoparticles ranging in size from 2 to 10 nm [148]. Optical and electronic properties of QDs differ from larger particles and are defined by quantum mechanics. QDs exhibit photoluminescence in the NIR region and can be used as a label to a targeting moiety. The small size of QDs is also beneficial in terms of tumor penetration. 

Decoration of Zinc sulfide (ZnS)-capped cadmium selenide (CdSe) QDs with RGD or LDV peptides resulted in their specific accumulation in HT1080 fibroblast sarcoma cells [119]. Other example is ZnCuInSe/ZnS core/shell QDs displaying the A20FMDV2 peptide for targeting α_v_β_6_-rich head and neck squamous cell carcinoma (HNSCC) [136]. This integrin is typically not detectable in non-pathological tissues, but is highly overexpressed on HNSCC. QD-A20 showed α_v_β_6_-specific binding in both two-dimension (2D) monolayer and three-dimension (3D) spheroid HNSCC in vitro models. The previous examples of peptide-decorated QDs showed emission at up to 700 nm, however a more red-shifted emission is desired. This region is preferred over NIR, as infrared irradiation allows for deeper tissue penetration. Coating silver sulfide (Ag_2_S) QDs with silica allowed for the shift in emission to 900–1200 nm [149]. The decoration of Ag_2_S QDs with Cilengitide resulted in vivo tumor visualization. Thus, integrin-binding QDs are suitable for cancer cell visualization both in vitro and in vivo with minimal UV-induced tissue damage.

The in vivo biodistribution profile of RGD-decorated ZnS/CdSe QDs was shown to be affected by the administration route in mice [150]. The predominant accumulation in liver was observed in both intravenous (i.v.) and intratumoral (i.t.) regimens, possibly due to QDs clearance. However, the i.t. injected RGD-decorated QDs retained in the SW1990 pancreatic tumor longer than non-targeted QDs or the i.v. injected targeted QDs. This was indicated by the Total Radiant Efficiency of the tumor area 24 h post-injection, which was three times higher in the former case. At the same time, 1/20 of the i.v. dose administered i.t. was enough to induce a comparable level of QD-mediated epi-fluorescence. No apparent in vivo cytotoxicity was observed regardless of the administration route [150,151].

Recently, the dual-targeting approach was also applied to QDs. CdTe QDs capable of binding both α_v_β_3_ integrin and gastrin-releasing peptide receptor were prepared via modification with two peptides, RGD and bombesin [124]. These QDs were shown to be highly selective for prostate cancer cells (PC-3) both in vitro and in vivo. The composition of the core QD allowed for dual modality as these QDs can be tracked using both PET and NIR fluorescence. Another example of dual targeting focused on EGFR and α_ν_β_3_ integrin [140]. These receptors bind two corresponding peptides, GE11 (sequence YHWYGYTPQNVI) and cyclo-(RGDfK). The decorated CdSe/ZnS QDs were able to distinguish cells with different expression patterns of the two receptors. Their cellular uptake efficiency was shown to be higher than that of a single-target QDs due to the synergistic effect of the two receptors on the uptake in HeLa cells.

An interesting approach to use QDs as part of a dual system for simultaneous imaging and PTT was reported by Zhang et al. [152]. Molybdenum disulfide (MoS_2_) nanosheets were stabilized with BSA and PEG, providing amino groups for conjugation to RGD and core/shell CdSSe/ZnS QDs. MoS_2_ was chosen as a NIR photosensitizer, and QDs as an imaging label. The resulting structures were approximately 4 nm thick and had submicron dimensions. These structures were colloidally stable in saline and cell culture medium, and did not exhibit cytotoxicity towards HeLa cells. Moreover, these composite structures were only targeting HeLa cells overexpressing α_v_β_3_, but not cells lacking this overexpression. In vitro and in vivo apoptosis of HeLa cells and tumor was observed as a result of PTT using these 2D structures. Furthermore, there were no signs of tumor recurrence 45 days post-treatment.

In summary, possible applications of such QD/integrin-targeting nanoprobes lie in the field of NIR bioimaging, imaging-guided surgery, and treatment response monitoring. Another application can be quantitative profiling of integrins expressed onto different malignant cells enabled by QD labeling as to reveal the phenotypic heterogeneity among different cancers or within the same type but at different stages of disease progression.

#### 5.2.2. Gold Nanoparticles 

AuNPs and MNPs are also extremely attractive for integrin-binding peptides display. These particles are capable of both presentation of multiple peptide molecules aiding efficient targeting, and conversion of imparted energy into heat. The latter causes cancer cell death as a result of so-called plasmonic photothermal therapy (PPTT) or magnetic hyperthermia therapy. In the course of such treatment, the local temperature in the tumor elevates to 40–47 °C (104–117 °F). Since AuNPs are plasmonic, upon irradiation with the laser wavelength matching their localized surface plasmon resonance (LSPR), a local temperature build-up sufficient to trigger cell death takes place [153,154]. In magnetic hyperthermia, MNPs are exposed to an alternating magnetic field inducing particles rotation. This imparted energy is converted into heat by MNPs due to energy dissipation and as a result cancer cell death occurs [155,156]. These alternatives to conventional photodynamic cancer therapy utilizing small molecule photosensitizers, are deemed to be safer and more well-tolerated by patients due to minimized off-targeting. It was suggested that magnetic hyperthermia and AuNP-mediated PPTT should be applied as part of a complex cancer treatment alongside radiotherapy or chemotherapy to achieve a synergetic effect. It has been reported that radiation treatment promotes cancer cell invasion through the activation of molecular mechanisms that involve integrins and fibronectin [157]. For this reason, selected examples of RGD- and cyclo-RGD-decorated AuNPs employed as radiotherapy sensitizers are also reviewed herein.

To date, only variants of the RGD peptide were used to decorate AuNPs (Table 2). The peptide conjugation to their surface allows for an increased avidity of the targeting peptide to the integrin receptor [125,158]. Distinct categories of integrin-targeting AuNPs can be concluded from published data: small gold nanoclusters (AuNCs below 5 nm in size), larger spherical AuNPs, and non-spherical particles such as gold nanorods (AuNRs) and gold nanostars (AuNSs). The size range is of importance as a particulate formulation below 5 nm in size is removed from the plasma through kidneys and excreted in urine. Thus, long-term accumulation of nanoparticles in the body and possible long-term toxicity are avoided. Even small vasculature inside solid tumors and micrometastases is accessible for these small AuNPs [158]. Additionally, AuNCs below 2 nm in size are fluorescent and can be used as radiotherapy sensitizers. However, AuNCs are not used in PPTT due to their low extinction. On the other hand, the typical position of the LSPR for AuNR and AuNS can vary between 600 and 1200 nm, in the NIR region of the electromagnetic spectrum, outside the region where water and biological molecules absorb. This is beneficial as such irradiation is not harmful to the healthy tissue and allows for deeper light penetration.

Targeting AuNCs modified with cycloRGD were evaluated for binding to their corresponding integrin [125], accumulation in tumor [159], and tumor growth inhibition in vivo [130]. It was shown that cyclic RGD preserved its functionality towards α_v_β_3_ when coupled to AuCNs, and this binding was enhanced by seven-fold due to multimerization of the peptide onto the AuCNs [125]. It should be noted that a short PEG_8_ was used to display the cyclic RGD peptide, which is potentially essential for proper recognition of the surface-bound peptide. This might explain the observed differences between similar targeting AuCNs equipped with a PEG spacer and AuCNs lacking of it reported by Poon et al. [159]. According to this study, accumulated concentration of the cyclic RGD-decorated AuCNs through a PEG spacer (5 kDa) in tumor was three-times that of the AuCNs conjugate without the spacer at 4 h post a single intravenous injection (i.v.). This emphasizes the importance of a spacer/linker choice for appropriate presentation and availability of bound integrin-targeting peptides. Another study showed that a single i.v. injection of cyclic RGD-decorated fluorescent AuCNs (no spacer) in 4T1 tumor-bearing mice (mammary carcinoma) was able to act as radiotherapy sensitizers and prevented tumor progression for at least 20 days, which is almost double in respect to radiotherapy alone [130]. However, cyclic RGD-decorated AuCNs alone could not prevent tumor growth despite the good level of accumulation in the tumor. Different levels of tumor accumulation of the different RGD-decorated AuNCs without a spacer might be related to size-dependent peptide presentation: 2.7 nm mean diameter showed low tumor accumulation while 1.7 nm—high tumor accumulation. To conclude, AuNCs have shown excellent tumor-targeting when conjugated to cyclic variants of RGD via a PEG spacer, however only fluorescent AuNCs are suitable for cancer therapy as radiosensitizers. From this standpoint, tumor-targeting AuNCs are very similar to tumor-targeting QDs.

Alternatively, it was shown that RGD-decorated AuNPs with the mean hydrodynamic size of 48 nm (29 nm Au core size) accumulate in H1299 tumor (murine model of human non-small cell lung carcinoma) to a much greater extent than 91 or 114 nm AuNPs with the same coating (51 or 80 nm Au core size, respectively) [160]. Approximately 15% of the i.v. injected dose of 48 nm RGD-decorated AuNPs was accumulated in the tumor. The plasma half-life of the 48 nm RGD-decorated AuNPs was found to be 35 min, which was 1.3-times higher than for the 91 nm AuNPs and 2.0-times higher than for the 114 nm AuNPs. The active targeting allowed for at least 3-times higher accumulation than that of non-targeting AuNPs of the same size. The binding of the RGD-decorated AuNPs was proven to be integrin-mediated in a competition assay with free RGD. These data serve as a proof that active targeting to tumors by means of integrin-targeting peptides indeed occurs for larger AuNPs. However, temporary kidney damage was suggested for all three size categories as a significant Au content was detected in urine 6 h post injection. These RGD-decorated AuNPs were designed for tumor angiogenesis targeted radiosensitization therapy, and unfortunately, were not tested in PPTT. 

Yang et al. proposed a strategy for a thorough assessment of targeting AuNPs in a pancreatic cancer in vitro model (single and multilayer, MIA-PaCa-2) prior to in vivo [161]. This approach allows to select best performing formulations for the next step of in vivo investigation. In this study, RGD-decorated AuNPs with the core size of 10 nm showed similar level of accumulation in the tumor as the above-mentioned study—10–12% of the injected dose (i.v.).

The El-Sayed group focused on the use of several peptide-modified AuNPs in PPTT in vitro and in vivo. They showed that spherical AuNPs with a 30 nm gold core decorated with (RGD)_4_-peptide and PEG-stabilized localized in the cytoplasm of HSC-3 cells after a 24 h incubation [162]. When AuNRs were used in the same setup, the cells were subjected to irradiation with two different lasers: a pulsed nanosecond laser (a single 6 to 7 ns pulse) and a continuous wave (cw) laser (duration of 5 min). It was found that the minimum energy at which cell death occurred was very different for the two lasers: 1 mJ for the pulsed nanosecond laser and 114 J for the cw laser. Cell death mechanisms depended on the laser type and dosage. Lower energy cw laser treatment led to apoptosis, while higher energy cw laser treatment led to necrosis. In the case of the pulsed nanosecond laser cancer cells underwent necrosis. Next, (RGD)_4_-AuNRs (average gold core size 25 by 6 nm) generated through the same coating procedure, were examined [131]. PPTT was again performed on HSC-3 using the cw laser (86 J, 1-min irradiation time). An exhaustive analysis showed that the cancer cell death did not occur at these laser and AuNRs doses, while certain disruptive processes in cell migration-associated cytoskeletal proteins were observed. This targeted PPTT mainly induced inhibition of Rho GTPases, actin, microtubule, and kinase-related signaling pathways. The authors suggested that these AuNRs could be extremely suitable for preventing and treating cancer metastasis in animals. Another study showed that AuNRs decorated with the same (RGD)_4_-peptide and nuclear localization signal peptide were capable of selective in vitro PPTT [163]. The authors exposed anti-tumoral M1 and protumoral M2 macrophages to the peptide-targeted AuNRs and observed selective laser heating and apoptosis of M2 due to a higher rate of their cellular uptake. Interestingly, M1 macrophages did not seem to be affected by this PPTT treatment.

In a study by a different group, PPTT by means of AuNRs displaying cyclic RGD was combined with DOX chemotherapy in vivo [164]. This combined treatment was administrated i.v. as a single dose and allowed human U87MG glioblastoma xenograft mice to survive over an experimental period of 48 days. However, free DOX, AuNR-bound DOX, PPTT with targeting AuNRs without DOX, or the same RGD- and DOX-bearing AuNRs without the PPTT treatment only ensured mice survival for 15–40 days. Similar results were obtained for AuNRs displaying cyclic RGD and loaded with DOX and paclitaxel [165].

Due to the irregular shape and high surface-to-volume ratio of AuNSs, they demonstrate excellent photothermal transduction efficiency as the electric field penetrates into these structures more easily compared to other shapes of AuNPs. AuNSs were also derivatized with RGD-based peptides [30,166]. AuNSs were coated with a conjugate of polymeric dendrimers and cyclic RGD and tested both in vitro and in vivo in a U87MG glioblastoma model [166]. I.t. injected RGD-bearing AuNSs showed high photothermal conversion efficiency producing a 24.2 °C increase in the tumor area temperature. A single treatment was sufficient to completely inhibit the tumor progression for at least two weeks. The other example, linear RGD-coated AuNSs were tested for targeted PPTT of hepatocellular carcinoma (HCC) both in vitro and in vivo [30]. It was shown that this treatment caused caspase-dependent apoptosis in HepG2 cells, signs of mitochondria-mediated apoptosis, loss of lysosomal membrane integrity, and enhanced autophagy. The AuNSs were administered i.v., no signs of significant toxicity were observed in vivo. Tumor temperature increased by ~20 °C during the PPTT treatment, comparably set up as in the above-mentioned study. Similarly, alongside the excellent bio-safety of this system, the tumor was ablated after a single treatment. However, integrin-targeting AuNSs are mainly employed for in vivo imaging [168] and Surface-Enhanced Raman Spectroscopy [169]. The common features observed for these nanoparticles include: high specificity and selectivity of the AuNSs binding to integrins, high precision (~6 nm) imaging of receptor-peptide interaction and hence tumor neovasculature as well as deep-seated tumors.

However, the use of AuNPs in cancer therapy development is not always accompanied by PPTT. Several reports suggest that AuNPs decorated with integrin-targeting peptides can markedly improve the outcome of a combined cancer therapy compared to a “solo” therapy [130,142,160]. For example, the administration of integrin-targeting AuNPs followed by γ-ray irradiation led to significant tumor progression inhibition due to radiotherapy enhancement [31,130,160]. A study on RGD-decorated AuNPs in radiosensitization therapy was conducted in different breast cancer models with a 20 nm core particle coated with PEG (5 kDa) and displaying (RGD)_4_ peptide [167]. Treatment of cancer cells with the RGD-AuNPs followed by ionizing radiation resulted in significant downregulation of fibronectin signaling by 50%. Judged by the invasion assay using Biocoat™ Matrigel^®^ Invasion Chambers, this phenomenon was accompanied by serious suppression of invasive activity by 67% compared to radiotherapy alone. This is an important finding for development of safer and more effective radiotherapy of highly invasive cancers.

Alternatively, AuNPs can be used as a solid core for the display or bioactive proteins with no irradiation or chemotherapy needed to perform the designed therapeutic effect. For example, 25 nm AuNPs were coated with Interleukin-12 (IL-12), an immunostimulatory cytokine known for anticancer and antimetastatic activity, and a conjugate of cyclic isoDGR and human serum albumin (HSA) via physi-/chemisorption to yield Iso1/Au/IL12 [142]. These dual AuNPs outperformed free IL-12 in murine models of fibrosarcomas and mammary adenocarcinomas, indicating that IL-12 is endowed with greater antitumor activity when presented onto these AuNPs, owing to an active targeting mediated by isoDGR peptide. Of note, one third of the mice treated with the dual AuNPs eradicated the tumor and resisted repeated challenge with the cancer cells to stay tumor-free for the whole course of the experiment (50 days). Moreover, these dual AuNPs were tested in the aggressive prostate adenocarcinoma mouse model alongside with adoptive T-cell therapy (ACT). Neither of the Iso1/Au/IL12 or adoptive T-cell therapies elicited significant anti-tumor effects when administered alone. However, co-injection of the two induced noticeable tumor regression though enhancement of effector functions of the tumor-specific T cells transferred in ACT. A follow-up study on murine fibrosarcoma model showed that isoDGR/Au/IL-12 AuNPs had a very similar therapeutic effect on the tumor volume to a treatment with free DOX, or to a combination therapy of the two [143]. Next, IL-12 was substituted for tumor necrosis factor (TNF). The combined treatment with a dual system isoDGR/Au/TNF and DOX lead to a significant tumor growth inhibition compared to free DOX. A triple system isoDGR/Au/IL-12+TNF alone was slightly less effective than free DOX, but a combined treatment with said AuNPs + DOX resulted in considerable tumor suppression. The synergistic effect was explained by affected endothelial permeability due to the isoDRG-peptide high-avidity targeting most likely followed by integrin-mediated cellular uptake, and consequential reduction in drug penetration barriers. Presence of the cytokines is important for triggering a reaction from immunocompetent cells directed towards the tumor. However, these data suggest that the displayed TNF played a more important role in the studied tumor (WEHI fibrosarcoma model) inhibition than the displayed IL-12. Alternatively, HSA was replaced with a PEG11-lipoamide spacer to display isoDGR [128]. This allowed to reduce molecular heterogenicity of this system with no apparent loss in its performance Nonetheless, the potential of the trifunctional iso1/Au/TNF + IL12 in inducing synergistic effects in combination with other chemo- or immunotherapies in other tumors should be studied.

#### 5.2.3. Magnetic Nanoparticles

Another class of nanoparticles commonly employed in biomedical applications is MNPs [170,171,172,173,174,175]. Hyperthermia therapy by means of MNPs and integrin-binding peptides provide a highly controllable way of eradicating tumor as MNPs can be potentially magnetically guided. Localized hyperthermia by means of MNPs has been also demonstrated to sensitize tumors to chemotherapy and radiation therapy [176]. Additionally, extensive research is being conducted in the field of magnetic resonance imaging by means of integrin-targeting MNPs [177,178].

Core-shell magnetic-AuNPs (15.4 nm in size) were coated with two peptide ligands, one known to trigger potent mitochondria-dependent apoptosis, the amphipathic tail-anchoring peptide (ATAP), and iRGD [29]. Thus, iRGD was intended to perform cancer cell targeting and integrin-receptor mediated endocytosis, and ATAP was intended to home the particles inside the cell to mitochondria in order to induce cancer cell death. Hyperthermia combined with magnetic delivery were used to test these hybrid particles for their apoptotic capability in glioblastoma (U87vIII) and breast cancer (MDA-MB-231) cells. The cancer cell viability was decreased by 10–50% when only magnetic delivery was used, and when hyperthermia was added—by 15–60% in respect to free ATAP depending on the particle concentration. Later, the same group suggested incorporating a reactive oxygen species (ROS) scavenger-inhibitor, diethyldithiocarbamate, alongside iRGD into the MNPs [179]. For this, a mesoporous silica shell was chosen to encapsulate the small-molecule inhibitor, and iRGD was conjugated onto the silica shell. Only when both the modified MNPs and exogenous ROS inducers were used (no hyperthermia treatment), glioblastoma cells (U87-EGFR-viii) underwent apoptosis. When additional hyperthermia was applied, it was found that the glioblastoma cells responded weaker than the breast cancer cells. The authors suggested that the brain cells are more resistant to ROS-mediated stress and have higher levels of antioxidants due to their nature. In an in vivo breast cancer model (MDAMB-231) the modified MNPs were administrated i.t. and the ROS-inducing agent was administrated i.v. After four weeks of weekly injections, an 80% decrease in tumor volume versus the control group was observed. It was also shown that the modified MNPs injected i.v. exhibit good tumor-homing.

However, obtaining more experimental data on the in vivo behavior of differently sized and shaped AuNPs displaying other integrin-binding peptides rather than RGD, is of high importance. Integrin-targeting MNPs have only been tested in model of glioblastoma or breast cancer. Other integrin-binding peptides and tumor models should be considered for hyperthermia therapy mediated with MNPs.

### 5.3. Selenium Nanoparticles

Selenium nanoparticles (SeNPs) are known for their selective anticarcinogenic effects [180]. It was shown that SeNPs enhanced pro-apoptotic genes expression in several cancer cell lines alongside with adaptive and pro-apoptotic signaling pathways of cellular stress response related to endoplasmic reticulum (ER), and hence, the unfolded protein response [181].

Combining this effect with gene silencing by means of small interfering RNA (siRNA) and active targeting by means of cyclo-RGDfC led to pronounced effects in vitro and in vivo in cervical cancer [182], colorectal cancer [183], and HCC models [184]. In the cervical cancer model, the target gene to be silenced was Derlin-1 [182], an ER membrane protein responsible for transportation of unfolded or misfolded proteins from ER lumen to cytoplasm. Derlin-1 is overexpressed in many cancers, including cervical cancer, and its expression is closely related to the migration and development of tumors. Targeting RGDfCSe@siRNA (70 nm in diameter) demonstrated greater uptake in HeLa cervical cancer cells in comparison to a “healthy” human cell line. Moreover, these targeting SeNPs showed faster siRNA release in the former case. Proved selectivity of gene silencing in HeLa cells and suppressed cell invasion resulted in apoptosis, aided by generation of ROS. After a three-week course treatment with targeting RGDfC-SeNPs@siRNA, a two-week period of no tumor progression was observed in xenograft mice, while the control formulation without the siRNA showed no significant retardation compared to saline. A similar SeNP system was used to silence KLK12 expression in a colorectal cancer, both in vitro and in vivo [183]. Kallikrein-related peptidases (KLKs) are deemed to be a marker of colorectal cancers and mediate proliferation and metastasis of cancer cells. Similarly, significantly greater particle uptake in HT-29 colorectal cancer cells relative to non-cancerous cells was observed for the targeted delivery of RGDfC-Se@siRNA (80 nm in diameter). For these SeNPs, the silencing and general mode of action in vitro was very similar to [182]: HT-29 apoptosis was accompanied by reduction of the mitochondrial membrane potential and enhancement of ROS generation. Importantly, the in vivo antitumor study showed the same two-week retardation in tumor progression when targeting RGDfC-Se@siRNA were used. Next, this targeting SeNP system was tested in an HCC model with intended silencing of Sox2 expression [184]. As previously used target genes, Sox2 was chosen due to its role in promotion of proliferation, migration, invasion, and tumorigenicity of cancer cells, especially in solid cancers, such as HCC, lung cancer, colorectal, and breast cancer. As before, these targeting SeNPs (75 nm in diameter) were capable of selective delivery of the siRNA to liver cancer cells. The observed in vitro and in vivo behavior repeated previous findings, although a shorter period of post-treatment retardation in tumor progression was observed (~10 days).

As a whole, these results indicate that RGDfC-Se@siRNA have a therapeutic potential in multiple cancer models.

### 5.4. Other Types of Nanoparticles

Another example worth mentioning is a nonconventional approach utilizing a bacteriophage as a carrier system [185]. Although phages are not considered a classical nanoparticle, they are nano-sized. In this study, a phage was used as both a cytotoxic cargo carrier and as a display surface for an integrin-binding peptide. For this, cyclo(-CRGDKGPDC-), or iRGD, a cyclic derivative of the RGD peptide capable of binding multiple integrins, was conjugated onto the surface of MS2 bacteriophage (23–28 nm in size) to enable targeted delivery of the thallium (Tl^+^) cargo MS2 was loaded with. According to the design, cellular uptake of this construct resulted in the release of the cytotoxic thallium load, followed by cancer cell death as evident from the in vitro data. iRGD-MS2-Tl^+^ construct exhibited a cytotoxic effect on both hormone-dependent and hormone-independent breast cancer cells (MCF-7 cells and MDA-MB-231, respectively). The in vivo data on MDA-MB-231 tumor in xenograft mice showed distinct tumor necrosis 12 days post-injection.

In the previous sections, nanoparticles with a multimodal or multifunctional ligands were described. However, there are many reports on the use of hybrid nanoparticles targeted by integrin-binding peptides in cancer research. A popular solution for combining integrin-targeted PPTT and encapsulation of small-molecule anti-cancer drugs is the use of a mesoporous silica coating onto the surface of AuNPs [186,187,188] or MNPs [179]. Polymer-based dendrimers can also be used for this purpose [166,189]. Alternatively, small-molecule anti-cancer drugs can be encapsulated into the hydrophobic core of polypeptide elastin-like nanoparticles, designed to display iRGD as part of their fusion polypeptide [190]. Combining magnetic and gold modalities facilitates delivery of the hybrid nanoparticles to cancer cells due to magnetic field and allows for hyperthermia therapy. At the same time, the gold shell can be readily modified with a variety of ligands, which protects the magnetic core from oxidation, and provides plasmonic properties [29]. Magnetic liposomes decorated with different RGD peptides were also reported [191,192]. More types of nanoparticles recently developed for integrin targeting by means of peptides include: protein-like [193], virus-like [194], and poly(lactic-*co*-glycolic acid) nanoparticles [195].

## 6. Conclusions

It has been previously suggested that integrin-binding peptides are outdated as therapeutic agents [196]. However, a large number of recent reports demonstrate that integrin-binding peptides are capable of guiding and “tumor-homing” different therapeutic nanoparticles varying in size (from 2 nm to several hundred nm), shape (spherical, rod-like, or star-like) and rigidity (lipid- or polymer-based, and solid metallic nanoparticles). At the same time, the development of new nanoparticle-based cancer theranostic formulations is lagging behind the discovery of new integrin-binding peptides by at least 10 years. Therapeutic effects of newly discovered integrin-binding peptides such as ATN-161, YCPDC, or A20FMDV2 are reportedly promising. However, their potential as a targeting moiety for nanoparticles has not been yet fully elucidated. The commonly used RGD peptides show affinity to integrins in the mid-nanomolar range, but some of the new peptides show sub-nanomolar affinity. This holds promise that the new integrin-binding peptides should show less off-targeting and higher specificity towards certain integrins overexpressed onto cancer cells. Therefore, better understanding and more thorough assessment of possible cross-reactivity of the targeting peptides, possible off-targets, effect of conjugation on preservation of the targeting ability are still open questions and are yet to be investigated.

As for integrin-targeting nanoscaled formulations, the influence of opsonization, clearance, as well as other events in the blood steam or tumor microenvironment are lacking and require urgent evaluation. These aspects need to be addressed in order to achieve successful in vivo translation of the reported promising result in vitro. PEGylation, the universal approach to prolong blood circulation of nanoparticles due to reduced opsonization, can cause peptide presentation issue and should be optimized on the basis of PEG molecular weight, coverage density or percentage of inclusion. The addition of a second targeting moiety for a plasma membrane-bound receptor, or capable of guiding the targeting nanoparticles inside the cell, has shown itself beneficial for PPTT or hyperthermia therapy. This is also fair for combination chemo- and radiotherapy. It was shown that many solid nanoparticles displaying integrin-binding peptides are guided to tumor integrins and sensitize the tumors in both cases. The authors of this review suggest that future efforts should concentrate on investigating integrin profiling for different cancer cells followed by comprehensive screening in in vivo cancer models. This would allow for new ways to discriminate between different cancers, as well as fight the metastatic disease and highly invasive cancers effectively by means of targeted PPTT, hyperthermia, or combination cancer therapy.

## Figures and Tables

**Table 1:** Known integrin-targeting peptides and their relation to integrin targets and different cancer types.

Peptide	Sequence ^1^	Binding Integrin	Cancer Type	Ref.
RGD, (RGD)_4_	RGD, RGDRGDRGDRGDPGC	α_v_β_1_, α_v_β_3_, α_v_β_5_, α_v_β_6_, α_v_β_8_, α_5_β_1_, α_8_β_1_, α_IIb_β_3_	Broad spectrum antagonist with sub-micromolar binding affinity	[21,22]
Cilengitide^® 2^	cyclo(-RGDfV-), end-to-end cyclized	α_v_β_1_, α_v_β_3_, α_v_β_5_	Supportive therapy for breast cancer therapy, glioblastoma, pancreatic cancer	[38,39]
iRGD (Internalizing RGD)	cyclo(-CRGDKGPDC-)-amide	α_v_β_3_, α_v_β_5_	Prostate, breast, pancreas, and colon cancers; glioma; bone metastasis	[40,41]
cyclo[RGDfK(C-ε-6-aminocaproic acid)]	cyclo(-RGDfK-), C-ε-6-aminocaproic acid coupled to the Lysine side chain	α_v_β_3_, α_v_β_5_	Pancreatic cancer and glioblastoma	[42,43,44,45,46]
cyclo-(-FRGDLAFpP-)	cyclo(-FRGDLAFpP-), end-to-end cyclized	α_v_β_6_	Colon, lung, cervix, breast, ovaries and fallopian tube, pancreas, head and neck cancers	[47]
cyclo-(-GLRGDLpP-)	cyclo(-GLRGDLpP-), end-to-end cyclized	α_v_β_8_	Human melanoma, glioblastomas, ovarian carcinomas	[48]
LDV	cyclo(-GLVD-BTD), cyclo(-SlDVP-), end-to-end cyclized, cyclo(-CWLDVC)	α_4_β_1_, α_4_β_7_	Leukemia, melanoma. Mainly used in infectious disease studies	[49,50,51,52,53]
ATN-161	Ac-PHSCN-NH_2_	α_5_β_1_, α_v_β_3_	Advanced solid tumors typical for breast cancer, prostate carcinoma, glioma; metastasis of colon cancer in liver	[54,55,56,57,58,59]
PhScN	Ac-PhScN-NH_2_	α_5_β_1_, α_v_β_3_	The same as its predecessor. Demonstrates lower IC_50_ in breast cancer model	[57]
ALOS-4	cyclo(-CSSAGSLFC-)	α_v_β_3_	Melanoma, glioma, prostate, and breast cancers	[23,60]
C16Y	DFKLFAVYIKYR	α_v_β_3_, α_5_β_1_	Ovarian cancer, breast cancer	[11,15]
YSNSG	cyclo(-YSNSG-)	α_v_β_3_	Solid tumors, melanoma	[61,62,63]
VLA4pep	YCPDC	α_4_β_1_	Multiple myeloma disease	[64,65]
GFOGER	GFOGER	α_2_β_1_, α_11_β_1_, α_1_β_1_, α_10_β_1_	Mainly used in cell adhesion studies of breast cancer cells	[18,66]
IKVAV	IKVAV	α_3_β_1_, α_6_β_1_	Mainly used in cell adhesion studies of breast cancer cells	[66,67,68]
AG86	LGGLPSHYRARNI	α_6_β_4_	HPV-18 positive cervical cancer	[69]
PR_b	SSPHSRNSGSGSGSG-SGRGDSP	α_5_β_1_	Prostate cancer	[19,70]
A20(FMDV2)	NAVPNLRGDLQVLA-QKVART	α_v_β_6_	Head and neck squamous cell carcinoma, pancreatic cancer	[71,72]

^1^ If not stated otherwise, the N- and C-termini are free of modifications. End-to-end cyclized peptides are accordingly indicated. Lowercase one-letter code for amino acids implies the presence of D-amino acids in the corresponding peptide sequence. C indicates that the peptide was cyclized via the side chain of cysteine residues. For the corresponding peptide structure, see Scheme 3 and Scheme 4. ^2^ EMD pharm. Merck KGaA.

**Table 2:** Summary of notable examples of integrin-targeting AuNPs.

Peptide	Type of AuNPs	Observed Effects	Ref.
cycloRGD	AuNCs (1.7–2.7 nm size range)	Higher affinity towards α_v_β_3_ was observed for targeting AuNCs. A PEG spacer between the peptide and the AuNC surface was crucial for enhanced tumor accumulation. However, no significant therapeutic effect was described, possibly due to the flawed NP design.	[125,130,159]
RGD	AuNPs (29, 51, and 80 nm in diameter)	The 29 nm AuNPs showed higher tumor accumulation, longer plasma half-life than larger AuNPs.	[160]
RGD	AuNPs (10 nm in diameter)	These smaller AuNPs showed similar tumor accumulation as the 29 nm AuNPs from [149].	[161]
(RGD)_4_	AuNPs (30 nm in diameter)	In in vitro PPTT cell death mechanisms depended on the laser type and dosage. Lower energy continuous wave (cw) laser treatment led to apoptosis, while higher energy cw laser treatment led to necrosis.	[162]
(RGD)_4_	AuNRs (25 by 6 nm)	This targeted PPTT mainly induced inhibition of Rho GTPases, actin, microtubule, and kinase-related signaling pathways.	[131,163]
cycloRGD	AuNRs (50 by 15 nm in [164]; 55 by 15 nm in [165])	These AuNRs were used for DOX or paclitaxel co-delivery. This strategy combined effects of chemo-phototherapy, active targeting of tumors, and multiple stimuli-responsive drug release.	[164,165]
cycloRGD	AuNSs	These AuNSs showed high photothermal conversion efficiency producing a 24.2 °C increase in the tumor area temperature. A single treatment was sufficient to completely inhibit the tumor progression for at least two weeks.	[166]
RGD	AuNSs	This treatment caused caspase-dependent apoptosis in HepG2 cells, signs of mitochondria-mediated apoptosis, loss of lysosomal membrane integrity, and enhanced autophagy. Tumor temperature increased by ~20 °C during the PPTT treatment, comparably set up as in the above-mentioned study.	[30]
(RGD)_4_	AuNPs (20 nm in diameter)	Combined radiotherapy with these AuNPs resulted in significant downregulation of fibronectin signaling by 50%. This phenomenon was accompanied by serious suppression of invasive activity by 67% compared to radiotherapy alone.	[167]
isoDGR	AuNPs (25 nm in diameter)	This multicomponent system comprised a gold core, cytokines IL-12 and/or TNF, and DOX. The synergistic effect was explained by affected endothelial permeability due to the isoDRG-peptide integrin-mediated uptake, and consequential reduction in drug penetration barriers. Presence of the cytokines was to trigger a reaction from immunocompetent cells directed towards the tumor. The results suggest that the displayed TNF played a more important role in the studied tumor inhibition than the displayed IL-12.	[128,142]

## Data Availability

Not applicable.
